# Effects of Whole-Grain and Sugar Content in Infant Cereals on Gut Microbiota at Weaning: A Randomized Trial

**DOI:** 10.3390/nu13051496

**Published:** 2021-04-28

**Authors:** Julio Plaza-Diaz, Maria Jose Bernal, Sophie Schutte, Empar Chenoll, Salvador Genovés, Francisco M. Codoñer, Angel Gil, Luis Manuel Sanchez-Siles

**Affiliations:** 1 Department of Biochemistry & Molecular Biology II, School of Pharmacy, University of Granada, 18071 Granada, Spain; jrplaza@ugr.es; 2 Instituto de Investigación Biosanitaria IBS. GRANADA, Complejo Hospitalario Universitario de Granada, 18014 Granada, Spain; 3 Children’s Hospital of Eastern Ontario Research Institute, Ottawa, ON K1H 8L1, Canada; 4 Research and Nutrition Department, Hero Group, 30820 Alcantarilla, Murcia, Spain; mjose.bernal@hero.es (M.J.B.); sschutte@hero.nl (S.S.); 5 Institute for Research and Nutrition, Hero Group, 5600 Lenzburg, Switzerland; 6 Biopolis-ADM, 46980 Paterna, Spain; Maria.Chenoll@adm.com (E.C.); Salvador.Genoves@adm.com (S.G.); fcodoner@gmail.com (F.M.C.); 7 Institute of Nutrition & Food Technology “José Mataix”, Biomedical Research Center, University of Granada, 18071 Granada, Spain; 8 CIBEROBN (CIBER Physiopathology of Obesity and Nutrition), Instituto de Salud Carlos III, 28029 Madrid, Spain

**Keywords:** infant cereals, infant food, whole grains, complementary feeding, intestinal microbiota

## Abstract

The introduction of complementary foods during infancy marks an important step in the development of the infant gut microbiome. Infant cereals are popular weaning foods but consistent evidence on their effect on the intestinal microbiota, especially when differing in nutritional quality, is lacking. Fecal samples from 4–7-month-old Spanish infants who consumed infant cereals differing in whole grain and sugar content as first weaning foods were analyzed on changes in microbial composition by massively parallel sequencing of the 16S ribosomal RNA gene at baseline and after 7 weeks of intervention. Samples were obtained from a previous trial conducted in Spain demonstrating whole-grain cereal acceptability. In total, samples of 18 infants consuming 0% whole grain cereals with 24 g sugar (0-WG) and 25 infants consuming 50% whole grain cereals with 12 g sugar (50-WG) were analyzed. Microbial composition changed significantly over time (*p* = 0.001), per intervention group (*p* = 0.029) and per infant (*p* = 0.001). Abundance of genus *Veillonella* increased in both groups while *Enterococcus* decreased. Within the 0-WG group, phylum Actinobacteria decreased along with genus *Bifidobacterium*. In the 50-WG, we observed an increase in *Lachnoclostridium* and *Bacteroides*. In addition, 50-WG decreased Proteobacteria and *Escherichia* to levels lower than 0-WG. Although weaning itself appeared to be responsible for most changes, the increased presence of anaerobic fermenters together with inhibition of pathogenic *Escherichia* may indicate a supporting effect of infant cereals with 50% whole grains and a reduced sugar content over infant cereals manufactured with refined hydrolyzed flours on the infant microbiota. In fact, using a novel methodology for the identification of microbial signatures, we found two groups of microbial taxa predictive of infants consuming enriched whole-grain infant cereals with a high predictive value of about 93%.

## 1. Introduction

Infancy is a crucial developmental period in which the foundations for optimal growth and development are established. The bacterial colonization in the infant gut during this period has received much review, as technological advances have allowed us to increasingly understand the pivotal role of the trillions of microorganisms inhabiting the intestinal tract [1]. Commensal intestinal microbiota can produce vitamins, improve nutrient digestibility, support normal intestinal motility, and, most importantly, can influence and stimulate the infant’s developing immune function [2,3,4,5,6,7]. Studies suggest that maintaining a healthy gut ecosystem could aid in preventing the early onset of several noncommunicable chronic diseases [2], while an overgrowth of specific microbial species or dysbiotic states can result in adverse effects in infants, such as faltering growth [8]. This stresses the need for a well-balanced microbiota composition. With very little intestinal microbes at birth [9], adequate bacterial gut colonization in early infancy is key.

Many factors have been described for their role in shaping the infant’s microbiota, such as mode of delivery, antibiotic use, genetics, and nutrition [10]. Human milk is the best nutrition for infants, containing unique factors that stimulate the pioneer gut microbiota including many different complex oligosaccharides and immunomodulatory components [11]. An increasing number of studies have investigated the role of early milk feedings. i.e., breastfeeding or formula, whether or not enriched with oligosaccharides, on infant’s fecal microbiota composition [12,13,14,15]. While the introduction of complementary foods is known to be a major factor contributing to further development and diversification of the microbiota, the effects of weaning are considerably less well studied [16].

Infant cereals are among the first foods introduced at the beginning of the complementary feeding period in many countries [17,18]. In Spain, infant cereals are often offered as first complementary foods [19]. These cereals are often based on wheat and rice [20] and the use of whole grains was not very common in Spain until very recently [21]. Cereals are an excellent source of energy, can easily be enriched with important nutrients such as iron, and their soft texture and smooth, semi-solid consistency might aid in transitioning from exclusive milk feedings to foods with increasing texture. Despite the major role of cereals during the weaning period, no consensus among pediatric organizations has been reached about adequate cereal intake; type of cereals, i.e., whole grain vs. refined cereals; or the degree of cereal processing [22]. Epidemiological studies have consistently demonstrated a beneficial relationship between whole-grain intake and risk of cardiovascular disease, obesity, and diabetes in adults [23]. However, infant cereals are usually manufactured with refined flours that are often hydrolyzed using amylases to limit their viscosity, which in turn increases the content of simple sugars [21]. In addition, other sugars such as sucrose are frequently added to infant cereals [24].

The differential health effects of refined versus whole grain, rich in fermentable and non-fermentable fibers and bioactive components, may, or at least partly, be conveyed via effects on the microbiota [25,26]. In addition, animal studies have linked excessive consumption of dietary sugars to detrimental functional changes in the gut microbiome [27,28]. However, information on the influence of cereal consumption on microbiota composition, especially in infants, is scarce [29]. Previously, we have demonstrated the sensory acceptability of cereals containing 50% whole grain and a medium sugar content (12 g/100 g) in 4–7-month infants, using a randomized 14 week cross-over design with 0% whole grain, high sugar (24 g/100 g) infant cereals as another treatment arm [30]. The objective of the present study is to compare the effects of infant cereals differing in nutritional quality on infant intestinal microbiota, using fecal samples collected at baseline, i.e., the beginning of complementary feeding, and after the first intervention period (7 weeks).

## 2. Materials and Methods

### 2.1. Ethical Statement

All patients enrolled in this study signed an informed consent form. The study followed the guidelines described in the Declaration of Helsinki and was approved by the ethics review committee of the “Hospital Universitario Virgen de la Arrixaca” (Murcia, Spain) (code: 2014–12–1–HCUVA). The study was registered at ClinicalTrials.gov NCT02781298.

### 2.2. Participants and Experimental Design

The present study is part of a larger study previously published to evaluate the acceptability of reduced sugar content and whole grain-based infant cereals [30]. Primary outcome of this study was the parent’s perception of infant’s reaction to the infant cereals. Power calculations for the previous study, was well as inclusion and exclusion criteria, are reported elsewhere [30]. Forty-nine healthy infants were recruited at seven primary health care centers that were allocated to the Health Area of “Hospital Universitario Virgen de la Arrixaca” in Murcia (Spain). The eligible infants had a gestational age of 37–42 weeks and a birth weight greater than 2500 g and were between 4 and 5 months of age. Furthermore, they were not breastfed since at least 4 months of age and not yet introduced to complementary foods. Children were randomly allocated to one of the two study’s branches using a random block system. The CONSORT checklist for a randomized clinical trial is available as supporting information (Supplemental Table S1). All following analyses were performed on a per-protocol basis.

### 2.3. Intervention Products

The two infant cereals used in this experiment were commercially available products from Hero España S.A (Murcia, Spain) based on wheat, corn, rice, oat, barley, rye, sorghum, and millet. The cereals needed to be prepared with infant formula. One of the two infant cereals contained 0% whole grain and a high sugar content produced by enzymatic hydrolysis (24 g/100 g) (0-WG). The other cereal contained 50% whole grain wheat flour and a low sugar content produced by enzymatic hydrolysis (12 g/100 g) (50-WG). The nutritional composition of the two infant cereals used in the present study is described in Table 1. Both infant cereals were provided in identical foil bags with the same label and were designed, produced, coded (two different production dates on the bag), and supplied by Hero España S.A.

### 2.4. Intestinal Microbiota Analysis

These analyses were conducted on a baseline fecal sample collected at the first visit of the study and a second fecal sample collected after seven weeks of intervention from each volunteer in plastic pots lined with a sterile plastic bag taken immediately to the laboratory by courier. Samples were stored directly at −80 °C. All analyses were conducted in Biopolis-ADM Genomics (Paterna, Valencia, Spain). DNA from fecal samples (baseline and seven weeks) was isolated using the procedure defined by Yuan et al. [31] with slight variations with the assistance of the MagnaPure Compact System (Roche Life Science) to prevent bias in the purification of DNA leading to Gram-positive bacteria misrepresentation. For massive sequencing, the bacterial 16S rRNA gene in the hypervariable region V3-V4 was amplified using key-tagged eubacterial primers [32] and was sequenced in a MiSeq Illumina using their recommendations (San Diego, CA, USA).

### 2.5. Bioinformatics

The resultant sequences were divided, considering the introduced barcodes during the PCR process. Reads from R1 and R2 files were introduced into the open-source software QIIME 2 2020.8 [33] using the input format with the q2-tools-import script through CasavaOneEightSingleLanePerSampleDirFmt. Reads were created using the V3-V4 16S rRNA sequencing. DADA2 [34] was utilized for denoising, operating an Illumina quality-aware model for amplicon errors to obtain an abundance distribution of sequence variances, which differ from a single nucleotide. After selecting quality results, q2-dada2-denoise script was employed to truncate forward reads at position 289 and trim at position 6. In the case of reverse reads, these were truncated at position 220 and trim at position 7. Removal of chimeras was made with the “consensus” filter via q2-dada2-denoise in that chimeras are identified in individual samples. MAFFT [35] was employed to align the amplicon sequence variants (ASVs) via q2- alignment and FASTTREE2 (via q2- phylogeny) [36] was used to construct the phylogeny. Sklearn naïve Bayes taxonomy classifier (via q2-feature-classifier) [37] was employed for creating taxonomy on ASVs using the SILVA 16S V3-V4 v132_99 [38]. Samples with less than 10,000 reads were excluded. Rarefaction curves were performed to determine whether the samples had been sequenced to an extent sufficient to represent their true diversity using the specaccum package from the last version of R (Supplemental Figure S1).

### 2.6. Assessment of Dietary Intake

Dietary data were collected using a two-nonconsecutive-day weighed dietary record [39,40]. The two dietary records were included in the parent’s questionnaires for food consumption and nutrient intake evaluation. The questionnaire was designed and validated in a previous study [41]. A comprehensive list of instructions was included in the parents’ questionnaires. Additionally, parents were instructed in a face-to-face interview on how to collect and provide a detailed description of the type of food consumed, time of day, ingredients used in recipes, prepared amount and ingested amount during two days (one weekday and another weekend day) before study visits after 1, 4, and 7 weeks of intervention. In addition, they could contact the pediatrician directly in case of any doubts. Data were transferred to an Excel file, and the types of foods consumed were clustered into 6 groups: milk formula, cereals, homemade meals, homemade fruits, commercial infant meals and commercial infant fruits. For homemade food (e.g., fruit purees or meals), parents were instructed to record the full recipe with raw materials (including amounts). For commercial infant foods, parents were asked to report the brand and the name of the product. Units of food/ingredients prepared and ingested were mainly collected in milliliters and grams. However, when measures were given in home measures such as spoons (table, dessert, or tea) or scoops, they were translated by the research team into exact weight according to tables of home measures in the Spanish population [42]. The nutritional profile for homemade baby foods was calculated with the use of the “United States Department of Agriculture (USDA) [43] Food Composition Databases” and the “Spanish Food Composition Database” (BEDCA) [44]. The nutritional information provided on the label was used for the calculation of the commercial infant foods. The dietary variables used in the present study were the amount of cereal consumed and the daily estimated intakes of energy, proteins, carbohydrates, total sugars, sugar from cereals, total fiber, fiber from cereals, fat and saturated fat. Dietary intake data were analyzed using an analysis of variance (ANOVA) for repeated measures, using ‘time’ (weeks) and ‘feeding group’ (cereal 0-WG or 50-WG) as fixed effects (SPSS version 27).

### 2.7. Statistical Analysis on Fecal Microbiota Composition

Descriptive quantitative variables are expressed as medians and ranges. Permutational multivariate analysis of variance (PERMANOVA) between cereal group, time, and infant, defined as the external variables, and the gut microbiome was achieved on 16S rRNA amplicon sequencing data of samples collected roughly between 0 and 7 weeks with the vegan R package, and the Adonis function. The external variable hierarchy in the PERMANOVA was performed by primarily analyzing each variable individually with a univariable PERMANOVA model and then ordering those variables established on the significance of their association (permuted *p*-value) since the highest to the minimum significant determinate in the PERMANOVA model. The statistical significance of the PERMANOVA results was evaluated by the permutation test with 999 permutations. Specific signatures on phylum and genus level were assessed using the Rivera–Pinto method and selbal algorithm; this method considers microbial signatures given by the geometric means of data from two groups of taxa whose relative abundances, or balance, are associated with the response variable of interest [45]. Principal coordinate analysis (PCoA) was used to identify clusters of samples with particular microbiota patterns; PCoAs were performed using open-source software QIIME 2 2020.8 with Bray–Curtis dissimilarity [33]. Alpha diversity (Shannon index) was assessed using the specaccum function, which finds species accumulation curves or the number of species for a certain number of sampled individuals, implemented for R version 3.2.3 [46]. Additionally, Simpson’s index, inverse Simpson’s index, richness species, and Pielou’s evenness index were measured with the R package vegan [47]. The number of species is a measure of richness—the more species present in a sample, the ‘richer’ the sample. Evenness is a measure of the relative abundance of the different species making up the richness of an area. As species richness and evenness increase, diversity increases. Simpson’s diversity index is a measure of diversity that takes into account both richness and evenness. The value of Simpson’s index of diversity (D) ranges from 0 to 1; the greater the value, the greater the sample diversity. Simpson’s index is usually expressed as its inverse (1/D), and the meaning is the same; the higher the diversity, the greater the value. The Shannon index was analyzed by using the QIIME microbiome pipeline [48]. Finally, the R corrplot package [49] was used to assess Pearson’s correlations between anthropometrics, demographics, dietary factors, and genus variables. The correlations were performed on baseline values for all infants and for values after 7 weeks for the 0-WG and 50-WG group separately. Pearson’s correlations are expressed for all variables, significant variables are highlighted in red (negatively correlated) or blue (positively correlated) and findings were corrected for multiple testing using the Benjamini–Hochberg procedure [50].

## 3. Results

### 3.1. Study Logistics and Subject Characteristics

**Figure 1:**
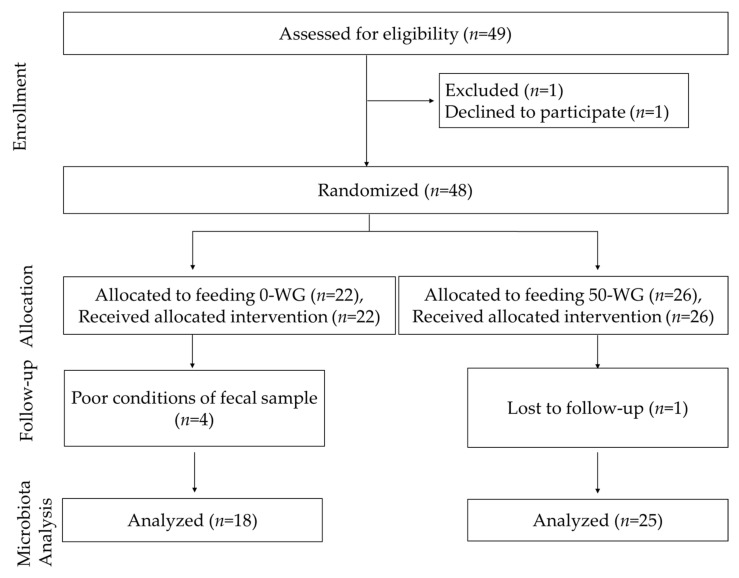
CONSORT flow diagram of the participants.

The study was carried out from September 2015 to September 2016. The total duration of the original cross-over intervention study was 14 weeks, both types of infant cereals were consumed for 7 weeks. For the present study, fecal samples from baseline, i.e., the beginning of complementary feeding, and after 7 weeks were used. Figure 1 depicts the CONSORT flow chart of the current study. Out of the 49 eligible infants, one declined to participate, and of the 48 infants enrolled in the study, 22 received 0-WG and 26 received 50-WG cereal. During the intervention period, one subject 50-WG dropped out by not attending the programmed visits. In total, the microbiota of fecal samples of 18 infants who consumed 0-WG and 25 infants who consumed 50-WG were analyzed, four samples from the 0-WG group were lost due to poor sample conditions. Baseline characteristics of the studied infants are shown in Table 2. There were no differences in age, birth weight, anthropometric variables, or milk feedings at baseline. All infants were formula-fed prior to the start of the study and not breastfed since at least 4 months of age. During the 7-week intervention period, there were no significant differences in infections and medication use between the two cereal groups (Supplemental Tables S2 and S3). All infants did not use antibiotics at least 15 days before enrolment and none used antibiotic treatments during the intervention period. One infant in the 0-WG group was reported to use probiotics in the 7th study week.

### 3.2. Changes in Dietary Intake during the Study Period

The dietary intake on macronutrient level as assessed on three study visits is shown in Table 3. During the 7-week intervention period, the total daily intake of protein (g), carbohydrates (g), and fiber (g) increased in both groups without significant differences between the two groups. However, the daily intake of fiber from the provided infant cereals (g) was significantly larger in the 50-WG group when compared to the 0-WG group during all study visits. In both groups, weaning from infant formula to cereals and other solid foods resulted in decreased daily intake of fat (g) and saturated fat (g). During the last two study visits (i.e., week 4 and week 7), total fat intake (g) was higher in the 50-WG when compared to the 0-WG group. Total daily energy intake (kcal) and daily sugar intake (g) did not significantly change during the intervention period, but the sugar content provided by the infant cereals was significantly lower in the 50-WG group compared to the 0-WG group at the study visits at 4 and 7 weeks.

### 3.3. Overall Effects of Introduction of Cereals as First Weaning Foods on Microbiota Composition

The considered factors for the PERMANOVA analysis i.e., group (0-WG vs. 50-WG), time of intervention (baseline vs. 7 weeks), and infant, were statistically significant (Group F: 2.1778; R^2^: 0.01775; *p*: 0.029; Time F: 7.2736; R^2^: 0.05928; *p* < 0.001; Infant: F: 1.7378; R^2^: 0.58068; *p* < 0.001) meaning that samples differed substantially among subjects as each infant had a unique microbial composition (about 58% of explained variance), but also to changes over time (about 6% of explained variance) and group (about 2% of the explained variance). Using PCoA, marginal separation in samples could indeed be observed when variance between samples was analyzed based on time (baseline vs. 7 weeks) (Supplemental Figure S2). Table 4 and Table 5 show the median and range of the predominant phyla and genera, respectively. Overlapping significant effects within both groups included an increase in the phylum Bacteroidetes as well as a decrease in the relative abundance of the genus *Enterococcus* along with an increase in the genus *Veillonella*.

### 3.4. Effect of Infant Cereals with Different Whole Grain and Sugar Contents on Microbiota Composition

As indicated earlier (see Section 3.3), treatment (0-WG vs. 50-WG) was shown to be a significant factor (*p* = 0.029) based on PERMANOVA analysis, but the microbiota variance explained was low (about 2%). PCoA showed large overlap in variance between intervention groups at baseline (Supplemental Figure S3) and we also found no significant differences between the groups on phylum and genus level, indicating an actual effect of intervention on microbiota composition. However, mixed effects were observed in the PCoA plot showing both the intervention groups and time (Supplemental Figure S4). The relative abundance of the main phyla and genera for 0-WG and 50-WG at baseline and 7 weeks are shown in Table 4 and Table 5, respectively. In the 50-WG group, the relative abundance of the phylum Proteobacteria decreased after 7 weeks (Table 4). Within the 0-WG group, the relative abundance of Actinobacteria decreased compared to baseline levels (Table 4). At the genus level, the relative abundance of *Escherichia-Shigella* was significantly lower at the end of the intervention in the 50-WG group when compared to 0-WG due a significant decrease in 50-WG (Table 5). In addition, genera *Lachnoclostrium*, *Bacteroides* increased within the 50-WG group while *Bifidobacterium* decreased within the 0-WG group (Table 5).

**Figure 2:**
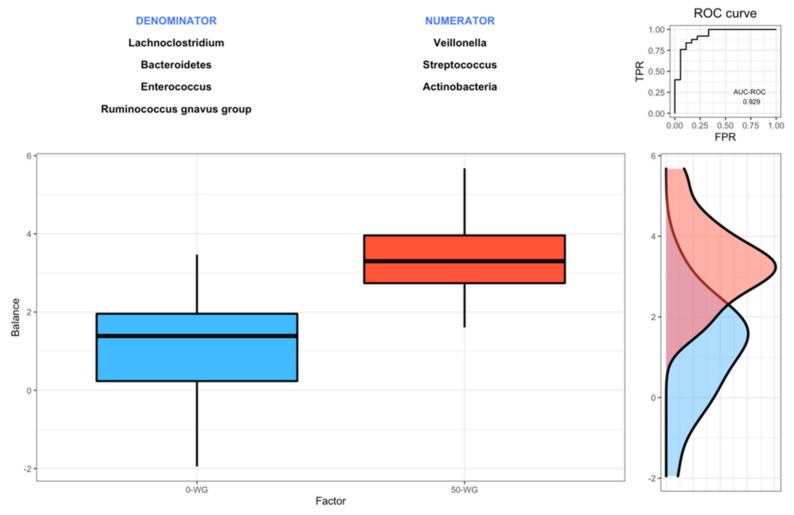
Description of the global balance for infants that received 0-WG and 50-WG cereals. The two groups of taxa and diversity indices that form the global balance are specified at the top of the plot. The box plot represents the distribution of the balance scores for 0-WG group and 50-WG group. The right part of the figure contains the receiver operating characteristic curve (ROC) with its area under curve (AUC) value (0.929) and the density curve for each group. TPR: True positive rate; FPR: False positive rate.

Figure 2 presents the distribution of the microbial signature values for 0-WG and 50-WG. Using the Rivera–Pinto method, we found two groups of taxa defining the microbial signature related to the type of cereal consumed, i.e., X_+_ = (*Veillonella*, *Streptococcus*, and Actinobacteria) and X_-_ = (Bacteroidetes, *Lachnoclostridium*, *Enterococcus*, and *Ruminococcus gnavus* group). Infants from 0-WG have lower balance scores than the 50-WG group, which means that the latter had lower relative abundances of *Veillonella*, *Streptococcus*, and Actinobacteria than Bacteroidetes, *Lachnoclostridium*, *Enterococcus*, and *Ruminococcus gnavus* group. The discrimination value of the identified balance was very high, with an area under the curve for the receiver operating characteristic curve (UC-ROC) value of 0.929, which means both groups can be accurately differentiated.

### 3.5. Effect of Infant Cereals with Different Whole Grain and Sugar Contents on Microbiota Diversity

**Figure 3:**
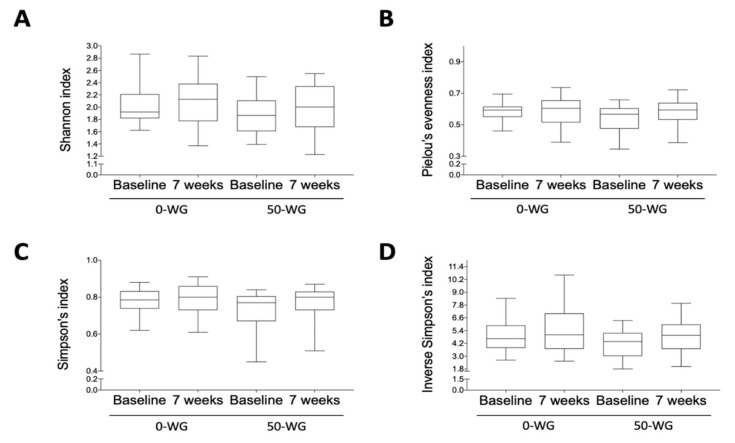
Alpha-diversity indices: (**A**) Shannon index, (**B**) Pielou’s evenness index, (**C**) Simpson’s index, (**D**) Inverse Simpson’s index. Boxplot values are means ±SEM, *n* = 18 per group for 0-WG and 25 for 50-WG.

No significant changes in Shannon index, Pielou’s evenness index, Simpson’s index, and inverse Simpson’s index compared to their respective baseline values were found after 7 weeks of intervention for both the 0-WG and 50-WG groups (Figure 3).

### 3.6. Correlations between Microbial, Anthropometric, Demographic, and Dietary Variables

Supplemental Figure S5 depicts the correlations among microbial (genus level), anthropometric, demographic, and dietary variables. At baseline in both groups, *Bifidobacterium* correlated negatively with *Ruminococcus gnavus* group. *Blautia* correlated positively with *Akkermansia* and negatively with sugars per day (Supplemental Figure S5A). *Akkermansia* correlated positively with the Shannon index. After 7 weeks of intervention, in the 0-WG group, we found that *Collinsella* and *Streptococcus* correlated positively with cereal consumption per day, sugars and fiber from cereal, and *Lachnoclostridium* correlated negatively with fiber per day (Supplemental Figure S5B). In the 50-WG group, we found that *Bifidobacterium* correlated negatively with *Escherichia-Shigella* (Supplemental Figure S5C).

## 4. Discussion

The main goal of the present study was to investigate the effects of two infant cereals with different whole-grain and sugar contents on the intestinal microbiota of healthy infants during weaning. To the best of our knowledge, this is the first attempt to evaluate the differential effects of the consumption of whole grain and reduced sugar infant cereal vs. a refined flour and high sugar cereal on the intestinal microbiota in early life. We observed high interindividual variation in fecal microbiota profiles of 4–7-month infants and a significant effect of time on the overall composition, meaning a shift during the study period most likely induced by the introduction of complementary foods, among which the infant cereals. Similar trends in changes in genera were observed for both cereals groups and differential effects between the whole grain sugar-reduced and the refined high-sugar infant cereals were modest. Nevertheless, using a novel methodology for the identification of microbial signatures [45], we found two groups of microbial taxa predictive of infants consuming enriched whole grain infant cereals with a high predictive value (0.929 for UC-ROC).

During the weaning period, the infant microbiota progressively diversifies and becomes more stable [10,51,52,53,54]. While increased protein and fiber intake are often mentioned as drivers for this diversification [54,55], we could not identify relevant dietary factors from the correlation analyses in our study. Transitioning to a more mature microbiota is suggested to have beneficial host effects such as increased vitamin biosynthesis and xenobiotic degradation, training and shaping of the immune system, and a growing ability to metabolize increasingly complex substances leading to the production of valuable fermentation products [40,41,53]. Previous studies on microbiota composition in the weaning phase show a replacement of dominant phyla Proteobacteria and Actinobacteria by the more adult-like phyla Firmicutes and Bacteroidetes [52,53]. Indeed, in both cereal groups, the abundance of Bacteroidetes significantly increased, whereas Firmicutes was the most dominant phylum at the end of the intervention. While Firmicutes were already dominant at baseline in the whole grain sugar-reduced cereal group, for the refined flour high-sugar group, this was only the case at the end of intervention when Firmicutes increased at the expense of a significant decrease in the early colonizing Actinobacteria. Only in the whole-grain cereal group, the abundance of Proteobacteria decreased to levels lower than in the refined flour group. Summarizing, the overall phyla shift appeared to be similar in both groups, potentially with a slight delay in the refined flour cereal group. However, as there were no significant differences in microbiota composition between the two groups at baseline, the observed effects might partially be attributed to whole grain cereals. For instance, De Filippo and colleagues showed that Burkina Faso infants exposed to a rural fiber-rich diet have significantly lower levels of Proteobacteria than their European counterparts who consumed significantly less fiber, [56] revealing a potential link between dietary fiber intake and a decrease in proteobacterial species.

Trait-based analyses on infant microbiome during the first three years of life indicate that microbial maturation over time is characterized, among others, by a decrease in oxygen tolerant species [57]. While the anaerobic phylum Bacteroidetes [58] increased in both groups, we mainly observed significant effects in the whole grain sugar-reduced cereal group on the genus level. In this group, relative abundances of the obligate anaerobic *Bacteroides* and *Lachnoclostridium* significantly increased. Indeed, the differences observed for the *Veillonella*, *Streptococcus*, and *Actinobacteria* on the one hand, and *Bacteroidetes*, *Lachnoclostridium*, *Enterococcus*, and *Ruminococcus gnavus* group, on the other, allowed us to predict infants consuming each type of cereal in an accurate fashion.

Infant cereals with increased content of whole grain and a reduced sugar content might support succession to a favorable oxygen-depleted habitat. Interestingly, *Bacteroides* and *Lachnoclostridium* harbor species that produce short-chain fatty acids (SCFAs), mainly butyrate to which favorable immunomodulating actions are ascribed [58,59,60,61]. It could be that the whole grain sugar-reduced cereals have increased SCFA-producing potential by providing fermentable fibers. Total fiber intake did not significantly differ between the cereal groups, but fiber from cereal was significantly higher in the whole grain sugar-reduced cereal group when compared to the refined flour high-sugar cereal group. Wheat bran contains β-glucans and arabinoxylans [62,63,64], which are not typically found in other non-cereal foods. A larger intake of these fermentable fibers could have stimulated the increase in saccharolytic genera. However, further research into the relative contribution of diet to the functional maturation of the infant microbiome is needed. For example, based on their findings, Koenig and colleagues argue that the infant microbiome is metabolically ready for receiving simple plant-derived foods, including cereals prior to the introduction of such foods [53], indicating that time, rather than diet, is a critical factor in the maturation process.

Declining milk feedings in the weaning period have been described to result in a decrease in bacteria involved in lactate metabolism that are anaerobic but oxygen tolerant [52,55,65]. Indeed, the major lactic and acetic acid-producing genera *Bifidobacterium* and *Enterococcus* decreased in both cereal groups, although the decrease in *Bifidobacterium* was only significant in the refined flour high sugar group. Bifidobacteria are described as gatekeepers in the succession process. An in-depth analysis on gut microbiota composition of Norwegian infants from birth to 3 years of age shows that the switch between children and adult community structure may be at least partially driven by the disappearance of bifidobacteria [66].

Another notable finding in our study is the decrease in Escherichia by the whole grain sugar-reduced cereals, resulting in significantly lower levels when compared to the refined flour high-sugar cereal group. This fast-growing genus contains several pathogenic species, some known to be involved in gastrointestinal diseases like diarrhea. While infants are particularly vulnerable to diarrhea, Vallès and colleagues showed that the changes and disturbances in the infant microbiome induced by solid foods did not per se facilitate an invasion of this opportunistic genus but rather a decrease in abundance [54]. However, we only observed a significant decrease upon weaning with whole-grain sugar-reduced cereals, indicating a supporting role of whole grains and/or lower sugar on pathogen inhibition. De Filippo’s trial comparing rural African with European infants also observed that high-fiber diets result in lower levels of *Escherichia*, leading them to conclude that the SCFA-producing bacteria, in particular, prevent the establishment of pathogenic species [56].

Infant cereals play an important role during weaning in numerous countries [17,19,67,68]. A reduction in sugar content and an increase in whole grains in infant cereals might be beneficial for infant health and could help to achieve recommendations for sustainable healthy diets [69,70]. Many national dietary guidelines support whole-grain foods as the primary choice of grain products in the diet [71,72,73,74,75,76,77,78]. It is expected that whole grain intake in infants could promote the long-term acceptance of whole-grain foods [22]. Previously, we reported that infant’s acceptance of whole grain [79] and whole grain sugar-reduced infant cereals [30] did not differ from their acceptance to refined flour high-sugar infant cereals. Lowering sugar intake in infants could have various health benefits such as the decreased risk of dental caries and childhood obesity as well as the adoption of healthier food preferences [80]. In contrast, infant cereals are commonly manufactured with added sugars, mainly sucrose, or contain sugars formed when starch is enzymatically hydrolyzed during processing, leading to the formation of glucose, maltose, isomaltose, and other low-molecular-weight saccharides [22]. Opposite to the proposed benefits of increasing whole grains in infant cereals is the concern of higher phytate levels in these types of cereals, which is associated with reduced mineral availability [81,82]. Previously, we have reported estimations on the phytate/mineral molar ratios in the intervention products [30], which were estimated not to compromise the availability of iron, calcium, and zinc. Additionally, a large Swedish trial showed that an extensive reduction in phytate content of infant cereals had no effect on growth, development or incidence of diarrheal or respiratory infections [83] and had little long-term effect on iron and zinc status [84]. In any case, iron intake in infants should be balanced. While infant cereals can be an important contributor to replenishing the infant’s depleting iron stores [85], there are indications that increased luminal iron levels negatively impact the microbiome, for instance by favoring pathogenic bacteria and by promoting gut inflammation [86,87]. As with many infant cereals on the Spanish market, the cereals in our study were enriched with iron. We observed no clear indications for a specific increase in pathogenic bacteria or susceptibility to bacterial infections in our study. Nonetheless, the complex relationships between iron status, health, and the microbiome in infants warrant further investigation. Prebiotic fiber GOS has been suggested to mitigate the adverse effects of iron fortification on the gut microbiome in infants [86,88]. This might also hold true for fiber types found in whole grains, which may have contributed to the protective effect of the whole grain sugar-reduced cereals on the growth of pathogenic *Escherichia*.

Summarizing, in this explorative study, we have observed modest differential effects of whole-grain sugar-reduced infants cereals compared to refined flour high-sugar cereals on the developing gut microbiome. Notwithstanding, the differences observed for the *Veillonella*, *Streptococcus*, and *Actinobacteria* on the one hand, and *Bacteroidetes*, *Lachnoclostridium*, *Enterococcus*, and *Ruminococcus gnavus* group, on the other, allowed us to predict infants consuming each type of cereal accurately. Strengths of the study include a relatively heterogeneous study population, as all infants were formula-fed, that received the intervention products as first weaning foods. Nonetheless, there are limitations to the present study. First, the study might not be sufficiently powered to detect all relevant microbial changes, as this was a secondary outcome, and whole grain content of the used infant cereals might be too low to exert relevant effects. Second, other foods were introduced during the study period that might have influenced the microbial composition, although there were no significant differences in macronutrient intake between the groups. Third, functional analyses are needed to establish potential metagenomic differences. Further research in this area is warranted.

## 5. Conclusions

Our study confirms that the transition of a milk-based diet to complementary foods alters the intestinal microbiota of infants. Furthermore, we observed a supporting effect of infant cereals with 50% whole grains and reduced sugar content as first weaning food over infant cereals manufactured with refined hydrolyzed flours on the fecal microbiota composition of 4–7-month infants, indicated by an increased presence of anaerobic fermenters along with a decreased abundance of the pathogenic *Escherichia*. However, a further in-depth research is needed to assess the benefits of whole-grain food consumption during infancy beyond increased acceptability, including their potential endorsing role in the functional maturation of the gut microbiome over time.

## Figures and Tables

**Table 1:** Nutritional composition of the two infant cereals.

Nutritional Content (Per 100 g)	0-WG	50-WG
Energy (kcal)	376	375
Protein (g)	9.8	10
Carbohydrates (g)	79.5	75
Sugars (g)	24	12
Fat (g)	1.3	2.0
Fiber (g)	4.0	7.2
Calcium (mg)	160	160
Zinc (mg)	6.23	8.16
Iron (mg)	0.63	1.09
Phytate (mg)	143.51	176.83

0-WG: 100% refined flour infant cereals with a high sugar content, 50-WG: 50% whole grain infant cereals with a low sugar content.

**Table 2:** Baseline characteristics of study subjects (infants).

	Intervention Group		*p*-Value
	0-WG *n* = 18	50-WG *n* = 25	
Gender infant *n* (%)			0.897
Female	9 (50%)	12 (48%)	
Male	9 (50%)	13 (52%)	
Birth weight (g)	3259 ± 339	3276 ± 446	0.898
Age (months)	5.16 ± 0.31	5.23 ± 0.41	0.496
Weight at inclusion (g)	7449 ± 909	7399 ± 789	0.850
Length (cm)	64.3 ± 2.9	65.3 ± 2.6	0.243
Head circumference (cm)	42.5 ± 1.5	42.6 ± 1.3	0.828
Formula-fed at inclusion *n* (%)	18 (100%)	25 (100%)	-
Type of formula:			0.416
Containing prebiotic fiber (GOS or FOS)	15 (83%)	20 (80%)	
Containing probiotics	2 (11%)	1 (4%)	
Without prebiotic fiber or probiotics	1 (6%)	4 (16%)	

Data are presented as mean ±standard deviation, 0-WG: 100% refined flour infant cereals with a high sugar content, 50-WG: 50% whole grain infant cereals with a low sugar content, GOS: galacto-oligosaccharides, FOS: fructo-oligosaccharides. Data were analyzed using Chi square test (type of formula) and Independent t-tests (all other variables).

**Table 3:** Changes in dietary intake on macronutrient level over intervention period (week 1, 4, and 7).

Dietary Factor	Intervention Group	Study Visit Week 1	Study Visit Week 4	Study Visit Week 7 End of Intervention
Infant cereal intake (g/100 g)	0-WG	28.9 ± 19.2	39.3 ± 20.8	34.5 ± 16.8
	50-WG	37.9 ± 34.7	38.3 ± 27.4	38.9 ± 24.9
Sugar from cereals (g)	0-WG	6.9 ± 4.6	9.3 ± 4.9	8.2 ± 4.1
	50-WG	4.7 ± 4.1	4.7 ± 3.2 ^#^	4.9 ± 3.9 ^#^
Fiber from cereals (g)	0-WG	1.1 ± 0.8	1.6 ± 0.8	1.4 ± 0.7
	50-WG	2.7 ± 2.5 ^#^	2.7 ± 1.9 ^#^	2.7 ± 1.7 ^#^
Energy (kcal/day)	0-WG	686.6 ± 190.9	683.3 ± 197.7	699.8 ± 188.5
	50-WG	760.4 ± 171.6	783.8 ± 223.7	797.7 ± 196.8
Protein (g/day)	0-WG *	14.7 ± 4.6	15.3 ± 5.7	17.7 ± 5.1
	50-WG *	15.9 ± 3.9	16.6 ± 5.8	20.1 ± 5.8
Carbohydrates (g/day)	0-WG *	89.2 ± 26.8	101.3 ± 31.1	107.7± 30.9
	50-WG *	97.0 ± 26.9	110.1 ± 38.0	118.3 ± 34.8
Sugar (g/day)	0-WG	60.8 ± 20.2	60.8 ± 20.9	59.8 ± 17.9
	50-WG	68.1 ± 17.7	68.4 ± 18.6	62.2 ± 16.3
Fat (g/day)	0-WG *	29.1 ± 9.1	23.9 ± 5.5	21.7 ± 6.6
	50-WG *	33.6 ± 7.5	29.9 ± 7.6 ^#^	26.9 ± 6.6 ^#^
Saturated fat (g/day)	0-WG *	12.4 ± 7.1	10.2 ± 6.2	8.6 ± 3.9
	50-WG *	13.1 ± 2.8	11.5 ± 2.9	9.8 ± 2.9
Fiber (g/day)	0-WG *	4.4 ± 3.8	5.4 ± 2.8	7.2 ± 1.9
	50-WG *	4.9 ± 3.2	6.2 ± 3.4	8.4 ± 4.1

Data are presented as mean ±standard deviation, 0-WG: 100% refined flour infant cereals with a high sugar content, 50-WG: 50% whole grain infant cereals with a low sugar content. Data were analyzed using repeated measures ANOVA with ‘time’ (weeks) and ‘intervention group’. * indicates significant difference (*p* < 0.01) in intake over time within an intervention group, # indicates a significant difference in intake (*p* < 0.05) between the intervention groups.

**Table 4:** Relative abundances (%) of bacteria in fecal microbiota of studied children at phylum level.

Bacterial Variables	0-WG		50-WG	
Phylum	Baseline (*n* = 18)	7 Weeks (*n* = 18)	Baseline (*n* = 25)	7 Weeks (*n* = 25)
Actinobacteria	47.5 (21.3–66.7)	33.2 (7.0–61.2) *	35.2 (2.2 -80.2)	34.9 (2.7–77.6)
Bacteroidetes	0.4 (0.0–6.1)	1.3 (0.0–26.6) *	0.6 (0.0–43.3)	2.3 (0.0–38.1) *
Firmicutes	29.5 (11.0–55.7)	33.3 (17.3–58.0)	39.1 (14.0–74.6)	43.4 (12.4–74.0)
Proteobacteria	15.2 (0.1–44.8)	21.6 (0.7–39.5)	15.2 (1.9–37.0)	10.3 (0.1–32.3) *
Verrucomicrobia	0.01 (0.0–19.3)	0.04 (0.0–34.7)	0.01 (0.0–20.6)	0.01 (0.0–6.7)
Species richness	30 (13–70)	35 (20–68)	34 (17 -56)	30 (18–63)
Sequences	88,539 (61,442–107,505)	93,989 (67,552–134,141)	88,521 (59,156–124,053)	86,305 (58,310–113,512)
Unclassified sequences derived from bacteria	0.0 (0.0–0.05)	0.04 (0.02–0.1)	0.03 (0.01–0.08)	0.04 (0.01–0.07)

Data are presented as median (range), table only shows phylum abundances with a value higher than 0.1%. 0-WG: 100% refined flour infant cereals with a high sugar content, 50-WG: 50% whole grain infant cereals with a low sugar content. Comparison between relative abundance at baseline and after intervention were analyzed using Mann–Whitney U tests, * indicates significant difference (*p* < 0.05) in relative abundance within an intervention group compared to baseline relative abundance.

**Table 5:** Relative abundances of bacteria in fecal microbiota of studied children at genus level.

Bacterial Variables	0-WG		50-WG	
Genus	Baseline (*n* = 18)	7 weeks (*n* = 18)	Baseline (*n* = 25)	7 weeks (*n* = 25)
*Bifidobacterium*	39.9 (7.5–66.3)	28.4 (6.6–59.2) *	28.4 (0.5–69.3)	28.7 (1.6–73.8)
*Escherichia-Shigella*	11.2 (0.0–41.4)	18.8 (0.1–39.1)	10.5 (1.3–34.5)	6.3 (0.06–28.4) *
*Blautia*	0.001 (0.0–3.9)	0.001 (0.0–11.9)	0.001 (0.0–7.6)	0.3 (0.0–1.9)
*Streptococcus*	1.5 (0.2–8.3)	1.3 (0.1–17.6)	2.2 (0.4–14.3)	1.5 (0.2–23.6)
*Enterococcus*	2.7 (0.3–20.5)	0.9 (0.0–9.3) *	1.5 (0.0–23.4)	0.6 (0.0–8.8) *
*Veillonella*	0.8 (0.0–16.1)	8.9 (0.0–25.7) *	4.4 (0.0–35.9)	9.3 (0.7–46.2) *
*Clostridium sensustricto* 1	0.2 (0.0–2.5)	0.9 (0.0–5.2)	0.4 (0.0–3.1)	0.5 (0.0–3.3)
*Eggerthella*	0.1 (0.0–1.2)	0.2 (0.0–0.9)	0.5 (0.0–1.4)	0.4(0.0–2.1)
*Granulicatella*	0.02 (0.0–1.0)	0.06 (0.0–0.2)	0.04 (0.0–0.6)	0.001 (0.0–0.4)
*Gemella*	0.02 (0.0–0.3)	0.04 (0.0–0.4)	0.09 (0.0–0.5)	0.1 (0.0–0.3)
*Lachnoclostridium*	0.04 (0.0–5.2)	0.3 (0.0–1.6)	0.001 (0.0–3.2)	0.7 (0.0–3.1) *
*Collinsella*	0.07 (0.0–30.0)	0.01 (0.0–25.6)	0.04 (0.0–27.2)	0.03 (0.0–20.9)
*Akkermansia*	0.01 (0.0–19.2)	0.04 (0.0–34.7)	0.01 (0.0–20.6)	0.01 (0.0–6.7)
*Ruminococcus gnavus* group	7.0 (0.0–31.1)	4.2 (0.0–27.0)	8.1 (0.0–61.0)	6.6 (0.0–29.6)
*Bacteroides*	0.3 (0.0–4.7)	1.2 (0.0–26.6)	0.2 (0.0–43.2)	2.1 (0.0–37.8) *
*Rothia*	0.002 (0.0–0.2)	0.002 (0.0–0.2)	0.004 (0.0–0.2)	0.003 (0.0–0.08)

Data are presented as median (range). 0-WG: 100% refined flour infant cereals with a high sugar content, 50-WG: 50% whole grain infant cereals with a low sugar content. Comparison between relative abundance at baseline and after intervention were analyzed using Mann–Whitney U tests, * indicates significant difference (*p* < 0.05) in relative abundance within an intervention group compared to baseline relative abundance.

## Data Availability

Raw FASTQ data are available upon request to the corresponding author.

## Supplementary Materials

The following are available online at https://www.mdpi.com/article/10.3390/nu13051496/s1, Figure S1: Rarefaction curves obtained for each of the sequenced samples, Figure S2: Principal Coordinate Analysis for all samples visualized by time (baseline i.e., 0 weeks vs. 7 weeks), Figure S3: Principal Coordinate Analysis for samples at baseline (0 weeks) visualized by intervention group. 0-WG: 100% refined flour infant cereals with a high sugar content, 50-WG: 50% whole grain infant cereals with a low sugar content, Figure S4: Principal Coordinate Analysis for all samples visualized by time (baseline i.e., 0 weeks vs. 7 weeks) and intervention group. 0-WG: 100% refined flour infant cereals with a high sugar content, 50-WG: 50% whole grain infant cereals with a low sugar content, Figure S5: Correlations between microbial, anthropometric, demographic, and dietary variables. Pearson correlations are expressed for all variables; significant variables are highlighted in red (negatively correlated) or blue (positive correlated) and corrected using the Benjamini–Hochberg procedure. (A) all infants at baseline i.e., 0 weeks (B) 0-WG after 7 weeks of intervention (C) 50-WG after 7 weeks of intervention. 0-WG: 100% refined flour infant cereals with a high sugar content; 50-WG: 50% whole grain infant cereals with low sugar content. Table S1: CONSORT 2010 checklist of information to include when reporting a randomized trial, Table S2: Frequency of infections and complications, Table S3: Frequency of treatments.
