# Supplementary material for: Effects of Whole-Grain and Sugar Content in Infant Cereals on Gut Microbiota at Weaning: A Randomized Trial
Source: Nutrients. 2021 Apr 28;13(5):1496. doi: 10.3390/nu13051496 (PMC8145071; doi:10.3390/nu13051496)
Supplement: Supplementary file 1 [file nutrients-13-01496-s001.zip › nutrients-1176136-supplementary.pdf]

Effects of whole-grain and sugar content in infant cereals on gut microbiota at weaning: a randomized trial

Julio Plaza-Diaz[1,2,3], Maria Jose Bernal[4,5], Sophie Schutte[4,5], Empar Chenoll[6,7], Salvador Genovés[6,7], Francisco M. Codoñer[6], Angel Gil[1,2,8,9,*] and Luis Manuel Sanchez-Siles[5,*]

[1]Department of Biochemistry & Molecular Biology II, School of Pharmacy, University of Granada, Spain. [2]Instituto de Investigación Biosanitaria IBS. GRANADA, Complejo Hospitalario Universitario de Granada, 18014 Granada, Spain. [3]Children's Hospital of Eastern Ontario Research Institute, Ottawa, ON K1H 8L1, Canada. [4]Research and Nutrition Department, Hero Group; 30820 Alcantarilla, Murcia, Spain. [5]Institute for Research and Nutrition, Hero Group, 5600 Lenzburg, Switzerland, [6]Health & Wellness-ADM Nutrition-ADM Lifesequencing, 46980 Paterna, Spain. [7]Health & Wellness-ADM Nutrition-ADM Biopolis, 46980 Paterna, Spain. [8]Institute of Nutrition & Food Technology "José Mataix", Biomedical Research Center, University of Granada, Spain. [9]CIBEROBN (CIBER Physiopathology of Obesity and Nutrition), Instituto de Salud Carlos III, 28029 Madrid, Spain.

*Author for correspondence: Angel Gil, Ph.D. Luis Manuel Sanchez Siles, Ph.D. E-mail: agil@ugr.es; luisma.sanchez@hero.es

Figure legends

Figure S1. Rarefaction curves obtained for each of the sequenced samples.

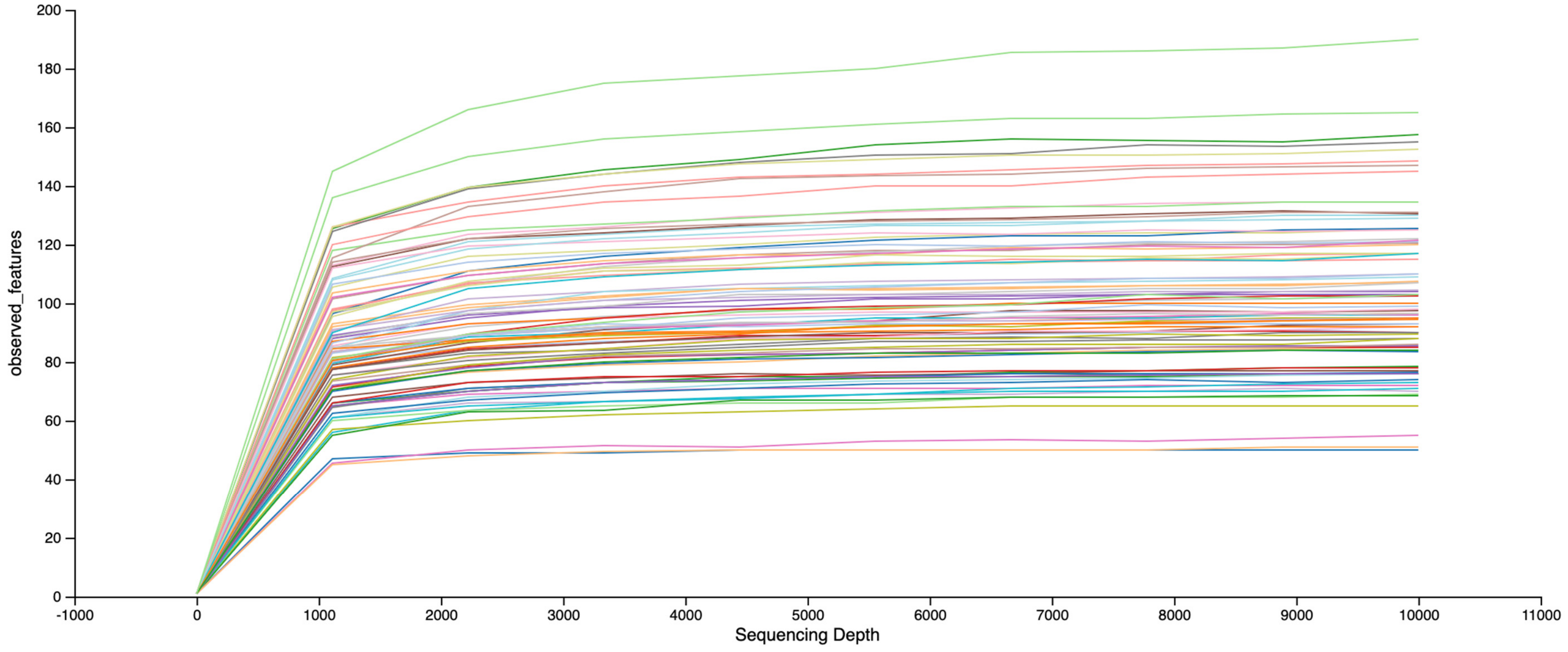

Figure S2. Principal Coordinate Analysis for all samples visualized by time (baseline i.e. 0 weeks vs. 7 weeks).

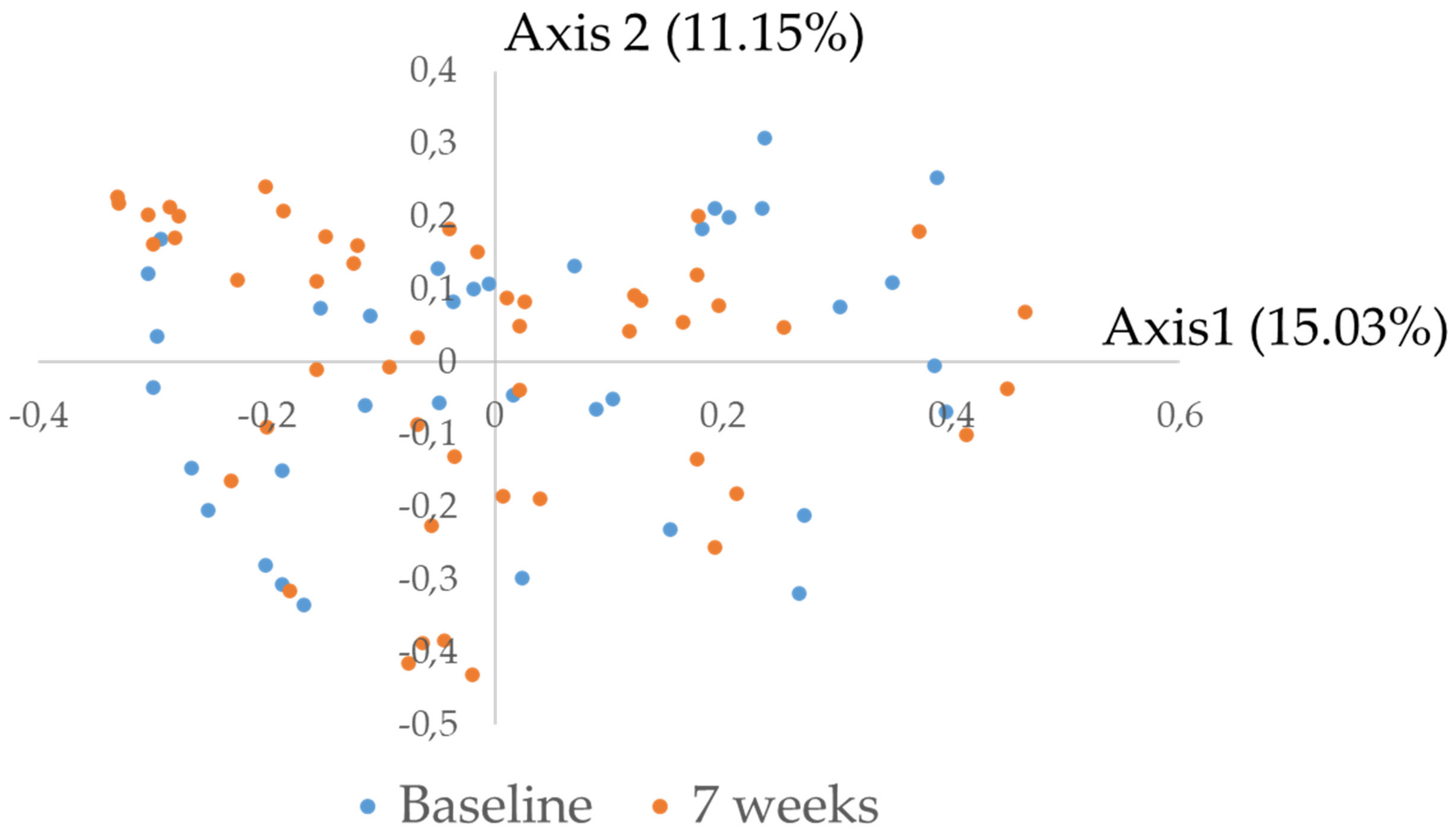

Figure S3. Principal Coordinate Analysis for samples at baseline (0 weeks) visualized by intervention group. 0-WG: 100% refined flour infant cereals with a high sugar content, 50-WG: 50% whole grain infant cereals with a low sugar content.

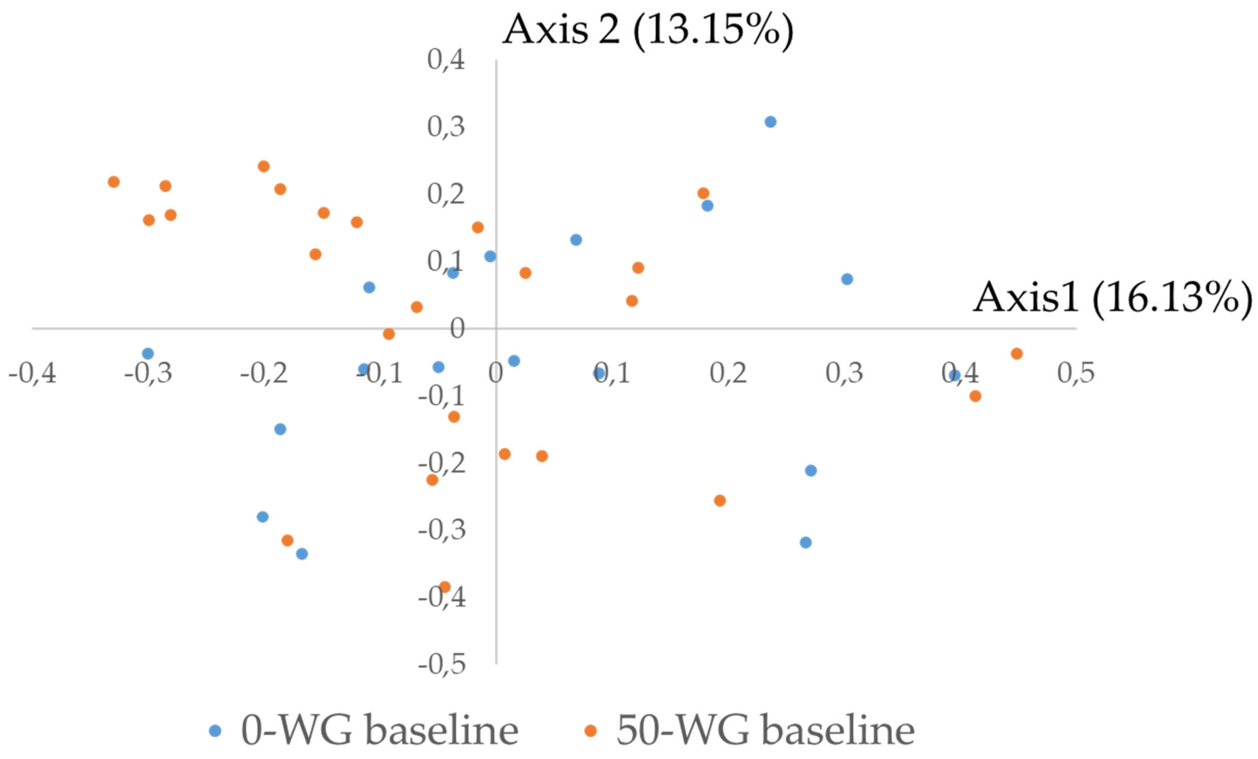

Figure S4. Principal Coordinate Analysis for all samples visualized by time (baseline i.e. 0 weeks vs. 7 weeks) and intervention group. 0-WG: 100% refined flour infant cereals with a high sugar content, 50-WG: 50% whole grain infant cereals with a low sugar content.

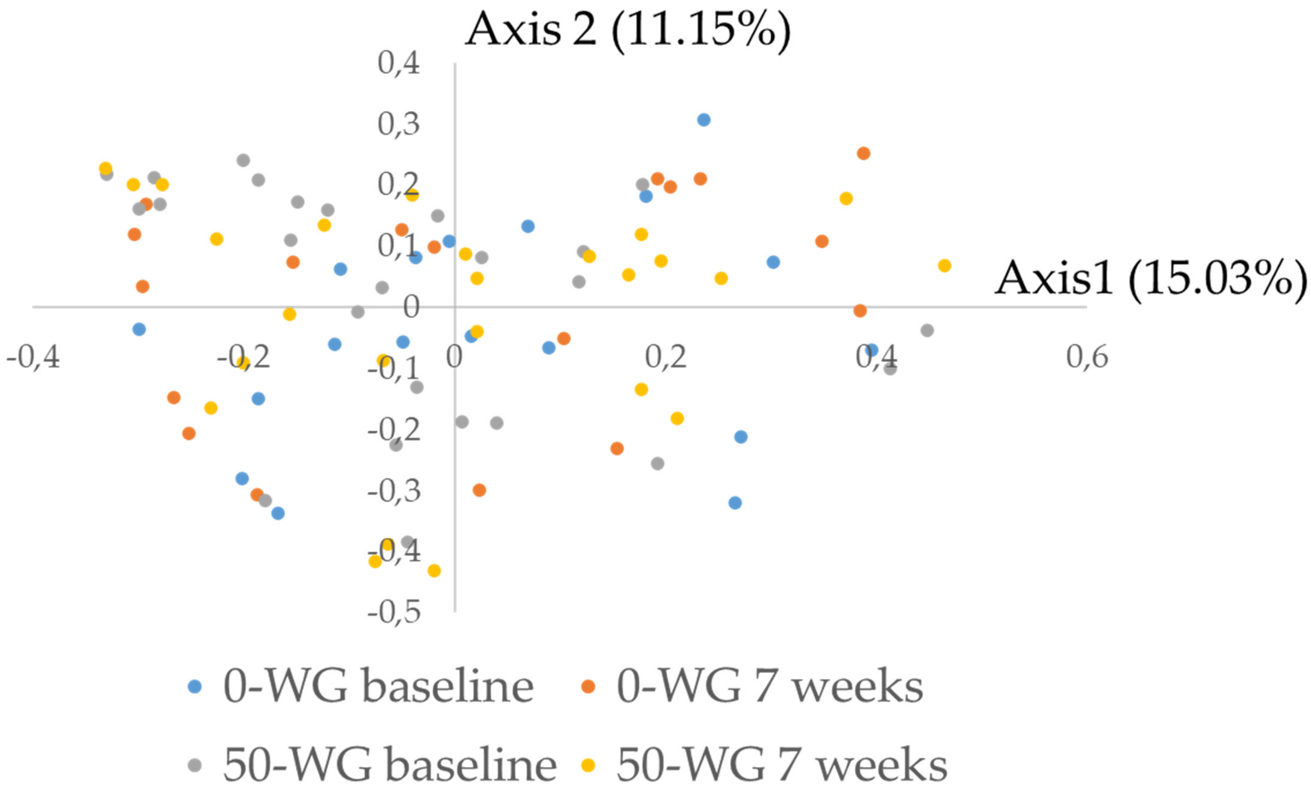

Figure S5: Correlations between microbial, anthropometric, demographic, and dietary variables. Pearson correlations are expressed for all variables, significant variables are highlighted in red (negatively correlated) or blue (positive correlated) and corrected using the Benjamini–Hochberg procedure. (A) all infants at baseline i.e., 0 weeks (B) 0-WG after 7 weeks of intervention (C) 50-WG after 7 weeks of intervention. 0-WG: 100% refined flour infant cereals with a high sugar content, 50-WG: 50% whole grain infant cereals with a low sugar content

A

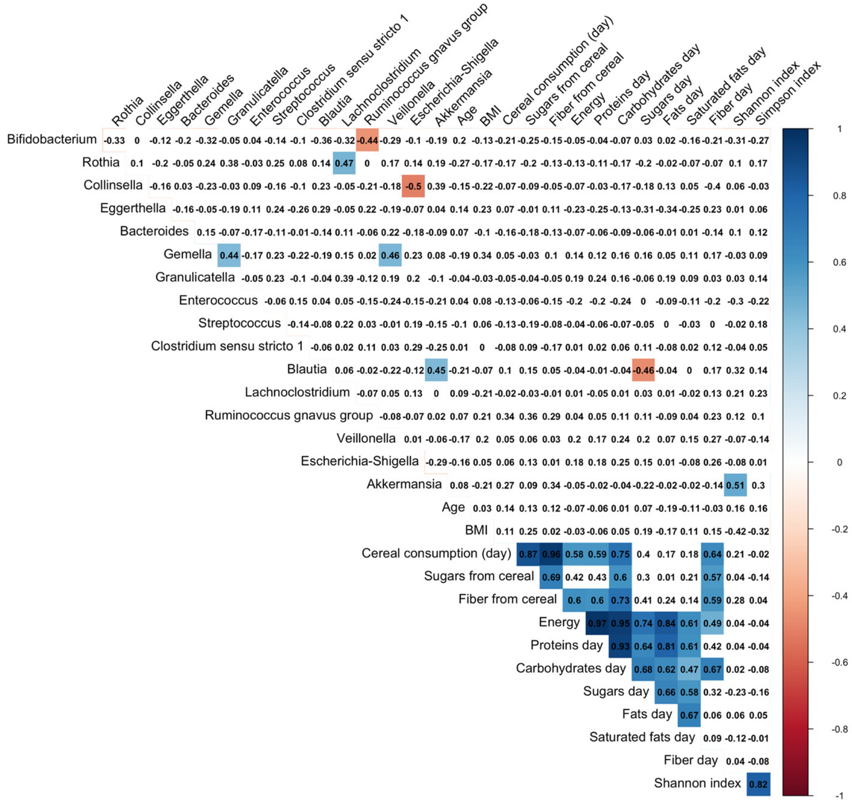

Bifidobacterium
Rothia
Collinsella
Eggerthella
Bacteroides
Gemella
Granulicatella
Enterococcus
Streptococcus
Clostridium sensu stricto 1
Blautia
Lachnoclostridium
Ruminococcus gnavus group
Veillonella
Escherichia-Shigella
Akkermansia
Age
BMI
Cereal consumption (day)
Sugars from cereal
Fiber from cereal
Energy
Proteins day
Carbohydrates day
Sugars day
Fats day
Saturated fats day
Fiber day
Shannon index
Simpson index
1
0.8
0.6
0.4
0.2
0
-0.2
-0.4
-0.6
-0.8
-1

B

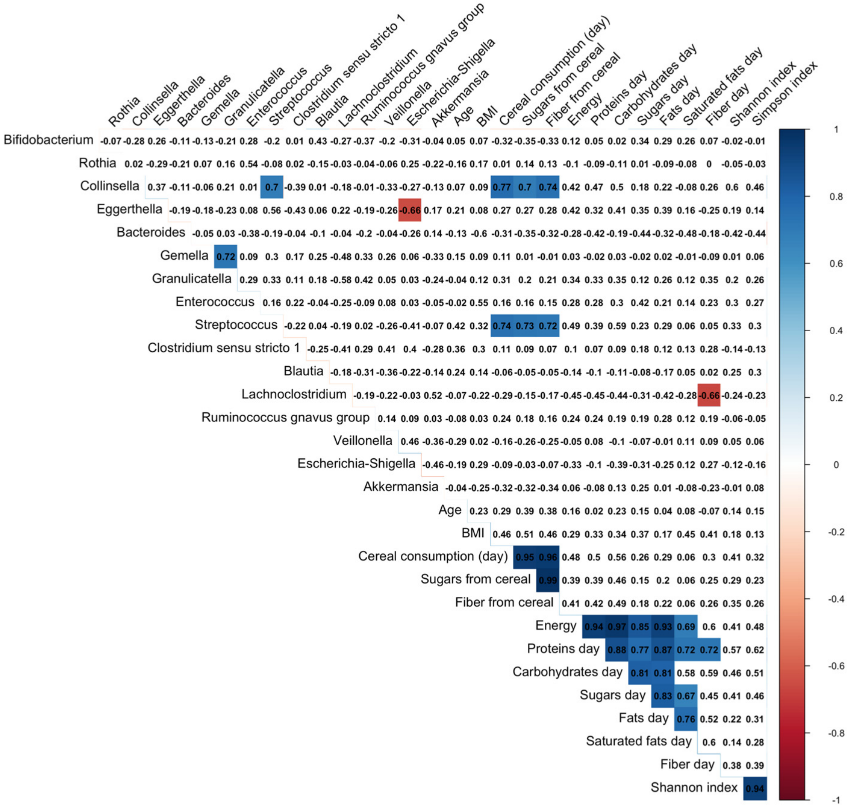
Rothia
Collinsella
Eggerthella
Bacteroides
Gemella
Granulicatella
Enterococcus
Streptococcus
Clostridium sensu stricto 1
Blautia
Lachnoclostridium
Ruminococcus gnavus group
Veillonella
Escherichia-Shigella
Akkermansia
Age
BMI
Cereal consumption (day)
Sugars from cereal
Fiber from cereal
Energy
Proteins day
Carbohydrates day
Sugars day
Fats day
Saturated fats day
Fiber day
Shannon index
Simpson index
Bifidobacterium
1
0.8
0.6
0.4
0.2
0
-0.2
-0.4
-0.6
-0.8
-1

C

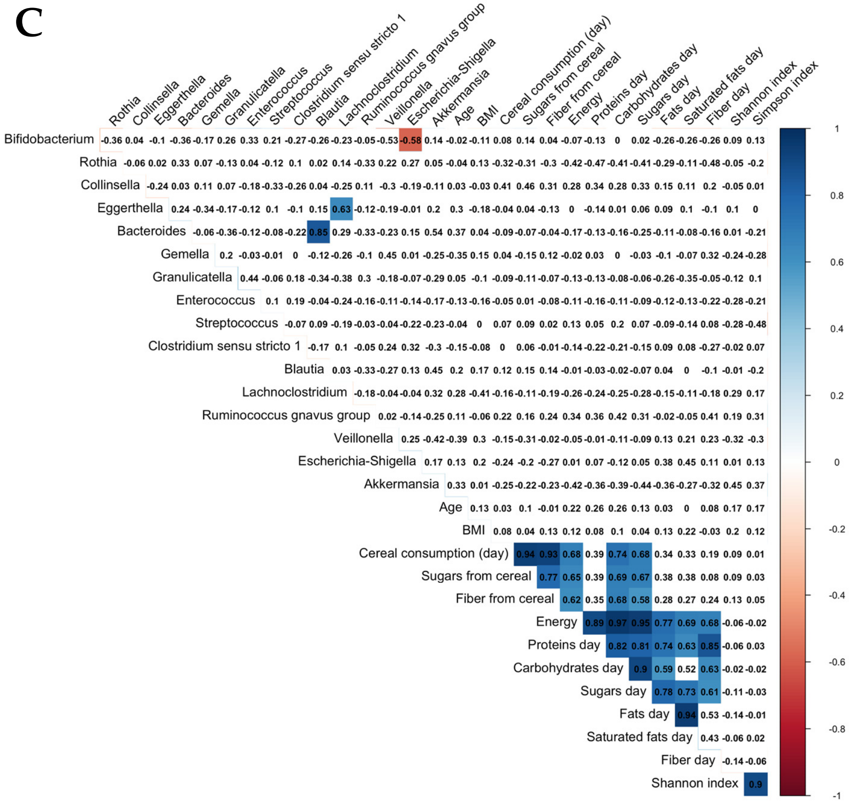
Rothia
Collinsella
Eggerthella
Bacteroides
Gemella
Granulicatella
Enterococcus
Streptococcus
Clostridium sensu stricto 1
Blautia
Lachnoclostridium
Ruminococcus gnavus group
Veillonella
Escherichia-Shigella
Akkermansia
Age
BMI
Cereal consumption (day)
Sugars from cereal
Fiber from cereal
Energy
Proteins day
Carbohydrates day
Sugars day
Fats day
Saturated fats day
Fiber day
Shannon index
Simpson index
Bifidobacterium
Rothia
Collinsella
Eggerthella
Bacteroides
Gemella
Granulicatella
Enterococcus
Streptococcus
Clostridium sensu stricto 1
Blautia
Lachnoclostridium
Ruminococcus gnavus group
Veillonella
Escherichia-Shigella
Akkermansia
Age
BMI
Cereal consumption (day)
Sugars from cereal
Fiber from cereal
Energy
Proteins day
Carbohydrates day
Sugars day
Fats day
Saturated fats day
Fiber day
Shannon index
1
0.8
0.6
0.4
0.2
0
-0.2
-0.4
-0.6
-0.8
-1

Tables

Supplemental material

Supplemental table 1. CONSORT 2010 checklist of information to include when reporting a randomized trial

Section/Topic	Item No	Checklist item	Reported on page No
**Title and abstract**			
	1a	Identification as a randomised trial in the title	1
	1b	Structured summary of trial design, methods, results, and conclusions (for specific guidance see CONSORT for abstracts)	1
**Introduction**			
Background and objectives	2a	Scientific background and explanation of rationale	1
	2b	Specific objectives or hypotheses	1
**Methods**			
Trial design	3a	Description of trial design (such as parallel, factorial) including allocation ratio	3 (+reported elsewhere)
	3b	Important changes to methods after trial commencement (such as eligibility criteria), with reasons	Not applicable
Participants	4a	Eligibility criteria for participants	3 (+reported elsewhere)
	4b	Settings and locations where the data were collected	3
Interventions	5	The interventions for each group with sufficient details to allow replication, including how and when they were actually administered	3+6 (Table 1)
Outcomes	6a	Completely defined pre-specified primary and secondary outcome measures, including how and when they were assessed	3
	6b	Any changes to trial outcomes after the trial commenced, with reasons	Not applicable

Sample size	7a	How sample size was determined	3 (+reported elsewhere)
	7b	When applicable, explanation of any interim analyses and stopping guidelines	Not applicable
Randomisation:			
Sequence generation	8a	Method used to generate the random allocation sequence	3
	8b	Type of randomisation; details of any restriction (such as blocking and block size)	3
Allocation concealment mechanism	9	Mechanism used to implement the random allocation sequence (such as sequentially numbered containers), describing any steps taken to conceal the sequence until interventions were assigned	3
Implementation	10	Who generated the random allocation sequence, who enrolled participants, and who assigned participants to interventions	3
Blinding	11a	If done, who was blinded after assignment to interventions (for example, participants, care providers, those assessing outcomes) and how	3
	11b	If relevant, description of the similarity of interventions	Not applicable
Statistical methods	12a	Statistical methods used to compare groups for primary and secondary outcomes	3+4+5
	12b	Methods for additional analyses, such as subgroup analyses and adjusted analyses	3+4+5
**Results**			
Participant flow (a diagram is strongly recommended)	13a	For each group, the numbers of participants who were randomly assigned, received intended treatment, and were analysed for the primary outcome	5 + Figure 1
	13b	For each group, losses and exclusions after randomisation, together with reasons	5 + Figure 1
Recruitment	14a	Dates defining the periods of recruitment and follow-up	5
	14b	Why the trial ended or was stopped	Not applicable
Baseline data	15	A table showing baseline demographic and clinical characteristics for each group	7 (Table 2)
Numbers analysed	16	For each group, number of participants (denominator) included in each analysis and whether the analysis was by original assigned groups	Not applicable (no missing variables for the final subset)
Outcomes and estimation	17a	For each primary and secondary outcome, results for each group, and the estimated effect size and its precision (such as 95% confidence interval)	8+9 (Table 4 & 5)
	17b	For binary outcomes, presentation of both absolute and relative effect sizes is recommended	8+9 (Table 4 & 5)

Ancillary analyses	18	Results of any other analyses performed, including subgroup analyses and adjusted analyses, distinguishing pre-specified from exploratory	5-11
Harms	19	All important harms or unintended effects in each group (for specific guidance see CONSORT for harms)	Not applicable
**Discussion**			
Limitations	20	Trial limitations, addressing sources of potential bias, imprecision, and, if relevant, multiplicity of analyses	13
Generalisability	21	Generalisability (external validity, applicability) of the trial findings	13
Interpretation	22	Interpretation consistent with results, balancing benefits and harms, and considering other relevant evidence	11-14
**Other information**			
Registration	23	Registration number and name of trial registry	14
Protocol	24	Where the full trial protocol can be accessed, if available	2 (ref 30)
Funding	25	Sources of funding and other support (such as supply of drugs), role of funders	14

CONSORT checklist derived from www.consort-statement.org

Supplemental table 2. Frequency of infections and complications

	Intervention group		p-value
	**0-WG** *n*=18	**50-WG** *n*=25	
**Study visit week 1**			
Nº episodes	14	22	0.405
Infections, *n*			
- Upper respiratory tract infections	3	2	
- Other infections including chicken pox	0	0	
**Study visit week 4**			
Nº episodes	16	21	0.454
Infections, *n*			
- Upper respiratory tract infections	2	2	
- Lower respiratory tract infections	0	2	
**Study visit week 7**			
Nº episodes	15	18	0.419
Infections, *n*			
- Upper respiratory tract infections	1	4	
- Other infections including chicken pox	0	2	
- Gastrointestinal infections	2	0	
- Non-infectious gastrointestinal symptoms (e.g. constipation, vomiting)	0	1	

Data are presented as absolute count, 0-WG: 100% refined flour infant cereals with a high sugar content, 50-WG: 50% whole grain infant cereals with a low sugar content. Data were analyzed using independent t-tests.

Supplemental table 3. Frequency of treatments

	Intervention group		p-value
	**0-WG** *n*=18	**50-WG** *n*=25	
**Study visit week 1**			
Nº treatments	16	22	0.929
Treatments, *n*			
- Antihistamines, anti-inflammatory, and bronchodilators	2	3	
**Study visit week 4**			
Nº treatments	16	21	0.301
Treatments, *n*			
- Antihistamines, anti-inflammatory, and bronchodilators	1	4	
- Antipyretics and analgesics	1	0	
**Study visit week 7**			
Nº treatments	16	17	0.417
Treatments, *n*			
- Antihistamines, anti-inflammatory, and bronchodilators	1	3	
- Antipyretics and analgesics	0	2	
- Probiotics	1	0	
- Laxatives	0	1	
- Inhaled or topical corticosteroids	0	1	

Data are presented as absolute count, 0-WG: 100% refined flour infant cereals with a high sugar content, 50-WG: 50% whole grain infant cereals with a low sugar content. Data were analyzed using independent t-tests.
